# Artificial Intelligence-based Approaches for Characterizing Plaque Components From Intravascular Optical Coherence Tomography Imaging: Integration Into Clinical Decision Support Systems

**DOI:** 10.31083/RCM39210

**Published:** 2025-07-29

**Authors:** Michela Sperti, Camilla Cardaci, Francesco Bruno, Syed Taimoor Hussain Shah, Konstantinos Panagiotopoulos, Karim Kassem, Giuseppe De Nisco, Umberto Morbiducci, Raffaele Piccolo, Francesco Burzotta, Fabrizio D’Ascenzo, Marco Agostino Deriu, Claudio Chiastra

**Affiliations:** ^1^Department of Mechanical and Aerospace Engineering, Polito^BIO^ Med Lab, Politecnico di Torino, 10129 Torino, Italy; ^2^Division of Cardiology, Department of Medical Sciences, Città della Salute e della Scienza, University of Turin, 10126 Turin, Italy; ^3^Department of Advanced Biomedical Sciences, University of Naples Federico II, 80131 Naples, Italy; ^4^Department of Cardiovascular Sciences, Fondazione Policlinico Universitario Agostino Gemelli IRCCS, Università Cattolica del Sacro Cuore, 00168 Rome, Italy

**Keywords:** intravascular imaging, optical coherence tomography, atherosclerotic plaque, artificial intelligence, machine learning, deep learning, automated plaque characterization, clinical decision support systems

## Abstract

Intravascular optical coherence tomography (IVOCT) is emerging as an effective imaging technique for accurately characterizing coronary atherosclerotic plaques. This technique provides detailed information on plaque morphology and composition, enabling the identification of high-risk features associated with coronary artery disease and adverse cardiovascular events. However, despite advancements in imaging technology and image assessment, the adoption of IVOCT in clinical practice remains limited. Manual plaque assessment by experts is time-consuming, prone to errors, and affected by high inter-observer variability. To increase productivity, precision, and reproducibility, researchers are increasingly integrating artificial intelligence (AI)-based techniques into IVOCT analysis pipelines. Machine learning algorithms, trained on labelled datasets, have demonstrated robust classification of various plaque types. Deep learning models, particularly convolutional neural networks, further improve performance by enabling automatic feature extraction. This reduces the reliance on predefined criteria, which often require domain-specific expertise, and allow for more flexible and comprehensive plaque characterization. AI-driven approaches aim to facilitate the integration of IVOCT into routine clinical practice, potentially transforming this technique from a research tool into a powerful aid for clinical decision-making. This narrative review aims to (i) provide a comprehensive overview of AI-based methods for analyzing IVOCT images of coronary arteries, with a focus on plaque characterization, and (ii) explore the clinical translation of AI to IVOCT, highlighting AI-powered tools for plaque characterization currently intended for commercial and/or clinical use. While these technologies represent significant progress, current solutions remain limited in the range of plaque features these methods can assess. Additionally, many of these solutions are confined to specific regulatory or research settings. Therefore, this review highlights the need for further advancements in AI-based IVOCT analysis, emphasizing the importance of additional validation and improved integration with clinical systems to enhance plaque characterization, support clinical decision-making, and advance risk prediction.

## 1. Introduction

Intravascular optical coherence tomography (IVOCT) is a catheter-based imaging modality that provides high-resolution cross-sectional images of the coronary arteries [1]. The IVOCT system uses a Michelson interferometer [2], which splits a broadband near-infrared light source into reference and sample beams to generate interference patterns for depth-resolved imaging. This technique detects backscattered and back-reflected signals, enabling high-resolution visualization of vascular structures, both at the surface and subsurface [3]. Intravascular image acquisition is performed using a probe mounted on a specialized catheter, which is inserted into the coronary artery and moved through a controlled pullback motion at a constant velocity. Since blood causes high backscatter due to light interaction with its particles, intermittent saline flushes are required during acquisition to maintain a blood-free imaging zone, ensuring optimal visualization of arterial structures. IVOCT is characterized by an excellent spatial resolution (10–20 µm) as compared to the typical imaging modalities used for coronary arteries, including conventional coronary angiography (100–200 µm), computed tomography angiography (400–700 µm), and intravascular ultrasound (IVUS, 100–200 µm) [4, 5]. IVOCT has a penetration depth of 1–2 mm, which may limit its ability to image deeper layers of the arterial wall, especially in the presence of highly attenuating structures [3]. Due to its unique characteristics, IVOCT enables the rapid observation of microscopic features in vascular cross-sections, making it a valuable tool for diagnosing coronary atherosclerosis [6]. The primary goal of IVOCT is to identify the edges of the vascular lumen and stent struts (if a stent is implanted). Additionally, image processing techniques enable the identification of atherosclerotic plaque features [7].

From a clinical perspective, IVOCT has several important applications. It enables the *in vivo* identification of plaque morphology such as thin cap fibroatheroma (TCFA), validates TCFA rupture, plaque erosion, and calcified nodules as key mechanisms of acute coronary syndromes, and differentiates between red and white thrombi [1]. Additionally, IVOCT (i) provides valuable insights into the temporal evolution of coverage in bare metal and drug-eluting stents, as well as bioresorbable scaffolds, (ii) supports the identification of neoatherosclerosis as a cause of late stent failure, and (iii) contributes to identifying vascular retraction as a toxic response to drug-eluting stents and stent malapposition [3, 8]. Consequently, IVOCT is increasingly used to guide percutaneous coronary interventions and is currently recommended with a Class IA recommendation by the latest European Society of Cardiology (ESC) guidelines in complex lesions [9]. Despite its high-resolution diagnostic capabilities, the impact of IVOCT on clinical practice remains limited. Challenges include the transition from IVUS, complex image interpretation, the lack of a standardized intervention guidance pipeline, and limited data from prospective clinical trials [3, 10].

In clinical practice, IVOCT is used for tasks such as measuring the minimum lumen area and minimum stent area, aided by the automatic annotations provided by the IVOCT systems software. However, for more complex analyses, such as plaque component categorization, cardiologists still rely on expert interpretation of IVOCT images, despite the detailed morphological information available. To address this, researchers have begun exploring artificial intelligence (AI) because of its high standards, reproducibility, and ability to process vast amounts of data [11, 12]. Some studies have focused on extracting features to automatically identify and classify different plaque types. However, systematic identification strategies and further research on plaque composition and characterization are still lacking [13].

Plaque regions in IVOCT images may be automatically characterized using traditional machine learning (ML) algorithms (like support vector machines, random forests, etc.) [14, 15, 16, 17]. Conventional ML approaches typically require multiple processing steps, including feature extraction, selection, and pixel classification. However, errors or inaccuracies at any stage can compromise the overall classification accuracy. An alternative approach involves deep learning (DL) techniques, which have gained popularity in medical image analysis [18, 19, 20, 21]. Among these, convolutional neural networks (CNNs) stand out for their ability to automatically and efficiently extract features from images, reducing the risk of errors in the feature extraction process.

This narrative review provides a comprehensive overview of AI-based methods applied to IVOCT images of coronary arteries for identifying atherosclerotic plaque components. The review is structured as follows: Section 2 describes key atherosclerotic plaque features detectable through IVOCT, including composition, microstructure, and rupture; Section 3 discusses state-of-the-art AI models for plaque composition characterization; Section 4 explores AI approaches for assessing plaque microstructure; Section 5 presents AI-based systems for detecting plaque rupture; Section 6 describes the most relevant AI-based systems for plaque characterization currently intended for commercial and/or clinical use; Sections 7 and 8 discuss the challenges and current limitations of these methodologies, propose potential solutions, and outline prospects for integrating AI into clinical decision support systems and risk prediction tools.

## 2. Atherosclerotic Plaque and its Components as Identified by IVOCT

**Fig. 1.**
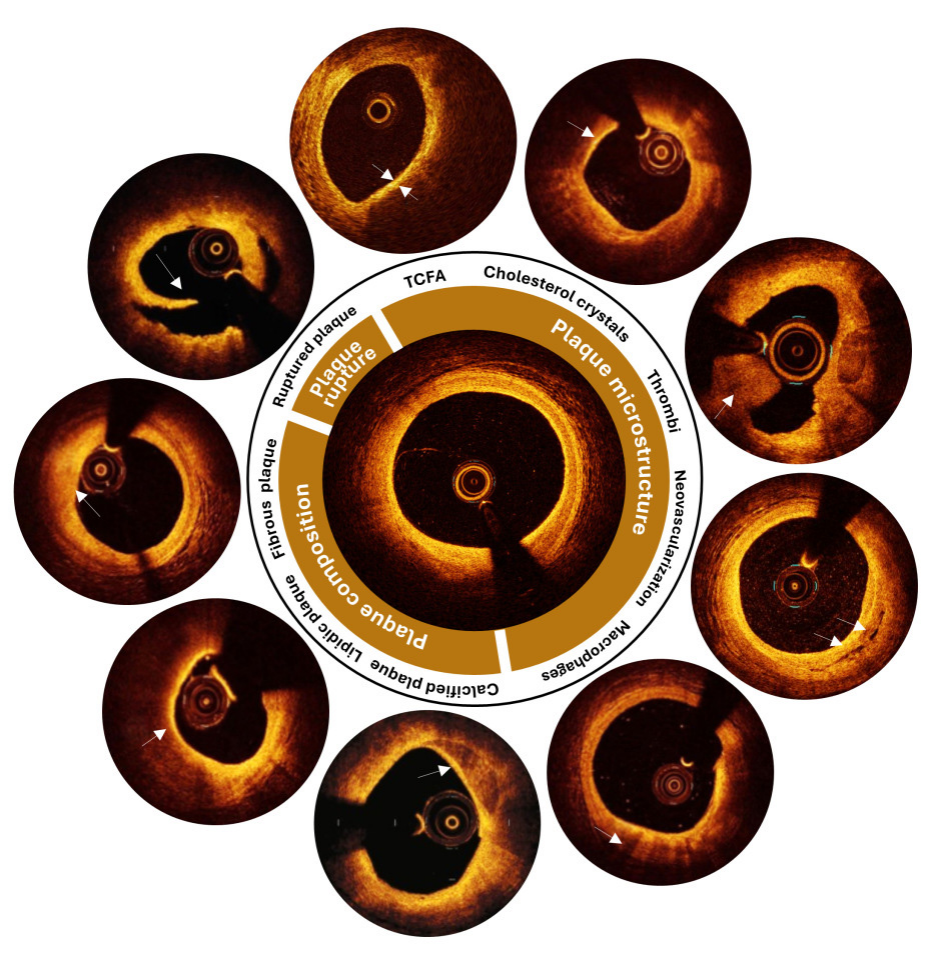
**Main components of an atherosclerotic plaque as visible in intravascular optical coherence tomography images**. Each plaque is characterized by its composition and microstructure, both of which can contribute to plaque rupture under certain conditions. The primary components of an atherosclerotic plaque include fibrous, lipidic, or calcified tissues. The microstructures commonly found in atherosclerotic plaques may include thin cap fibroatheroma, cholesterol crystals, thrombi, neovascularization, and macrophages. Image features are indicated by white arrows. Adapted with permission from [22, 23, 24]. TCFA, thin cap fibroatheroma.

As previously mentioned, due to its excellent spatial resolution and despite limitations in penetration depth, IVOCT enables the visualization of atherosclerotic plaques within the arterial wall of coronary arteries, allowing for the analysis of plaque components, microstructures, and rupture (Fig. 1, Ref. [22, 23, 24]) [6]. This facilitates the identification of high-risk features such as plaque layering, spotty calcification, the presence of lipids, thin fibrous caps, macrophage infiltration, microchannels, and cholesterol crystals, all of which are recognized as predictors of rapid plaque growth, and determinants of biomechanical stress [25]. These plaque features have been associated with the occurrence of major adverse cardiovascular events such as myocardial infarction and sudden cardiac death, even if the role they play remains to be further investigated [26].

This section describes the appearance of atherosclerotic plaques in IVOCT images, with specific reference to plaque components, microstructures, and rupture. The description is based on the consensus document prepared by the International Working Group for IVOCT Standardization and Validation (IWG-IVOCT), which establishes standard nomenclature, usage guidelines, image interpretation criteria, measurement methodologies, and reports recommended practices for interpreting coronary IVOCT results [1].

### 2.1 Plaque Composition

Lipid plaques appear in IVOCT images as low-signal regions with poorly defined borders (Fig. 1) [27, 28]. These plaques are more prevalent in cases with severe stenosis [29]. Lipid-rich plaques that are not treated with stenting often exhibit an unfavorable natural history, contributing to disease progression and an increased risk of adverse cardiovascular events [30, 31, 32, 33]. The presence of lipids within stented segments has been associated with in-stent plaque prolapse and intra-stent thrombus [34].

Fibrous plaques are characterized by a high-backscattering, slightly homogeneous IVOCT signal (Fig. 1), with occasional identification of the internal elastic lamina and external elastic lamina [1]. If elastic membranes cannot be identified, caution is advised when attempting to define this type of lesion [1]. Collagen or smooth muscle cells may also be observed in fibrous plaques using IVOCT [1]. Occasionally, due to the limited penetration depth of IVOCT, necrotic cores or calcium behind fibrous tissue may not be identified [1]. IVOCT allows clinicians to obtain precise measures of fibrous cap thickness, which is a significant predictor of plaque vulnerability [35].

Calcified plaques are characterized by well-defined, low-backscattering heterogeneous zones in IVOCT images (Fig. 1) [1]. They are often marked by superficial calcification at the affected region, without signs of burst lipid plaque. Calcification is a hallmark of advanced atherosclerosis, and its presence and severity are significantly associated with poor prognosis [36]. Additionally, small calcium deposits may contribute to the destabilization of lipid plaques [37].

### 2.2 Plaque Microstructure

A TCFA is typically defined as a necrotic core of an atherosclerotic plaque, delineated by an overlying thin fibrous cap with a minimum thickness below a predefined threshold (usually 65 µm) (Fig. 1) [1]. The necrotic core can be visualized in IVOCT images as a signal-poor region within an atherosclerotic plaque, with poorly outlined boundaries, rapid IVOCT signal drop-off, and minimal or no signal backscattering within a lesion covered by a fibrous cap. The fibrous cap is generally visible as a signal-rich tissue layer overlying a signal-poor region. To define TCFA, some studies specify that the necrotic core should cover more than one quadrant of an image in Cartesian coordinates or span an arc greater than 90° [1]. Delineating TCFA in IVOCT images requires caution, as artifacts can easily be misinterpreted as necrotic cores [1]. Previous research on the natural history of coronary lesions revealed that the presence of a TCFA is an independent predictor of future unfavorable cardiac outcomes [38, 39].

Cholesterol crystals appear as thin, linear regions of high intensity, typically associated with a fibrous cap or necrotic core (Fig. 1) [1]. Previous research revealed a possible link between clinical metabolic disorders and the presence of cholesterol crystals in patients with stable and unstable coronary syndromes [40].

Thrombi are visualized in IVOCT as masses attached to the luminal surface or floating within the lumen (Fig. 1) [1]. IVOCT distinguishes between two forms of thrombi: red thrombi, which exhibit marked backscattering and high attenuation, and white thrombi, which is less backscattering, homogenous, and has low attenuation. Thrombi can sometimes be mistaken for small dissections or intimal ruptures. For example, a red thrombus may be misdiagnosed as a necrotic core fibroatheroma. Additionally, thrombi may cause shadowing or obscure underlying structures. By recognizing thrombi, IVOCT is effective in identifying culprit lesions and understanding the underlying mechanisms of acute coronary syndrome [41].

Macrophage accumulations can be detected by IVOCT as signal-rich, discrete, or confluent punctate regions (Fig. 1), which are more intense than the background speckle noise. These findings should be evaluated only in the context of a fibroatheroma, as no macrophage validation studies have been reported for normal vessel walls or intimal hyperplasia [1]. Macrophages are often found close to the junction of a necrotic core and its cap, and their accumulation may sometimes be mistaken for microcalcification, cholesterol crystals, internal elastic lamina, or external elastic lamina. Macrophage accumulations may also attenuate IVOCT light, resulting in surface macrophages to shadow deeper tissue, giving a necrotic appearance [1]. A recent study demonstrated that macrophage infiltration at the culprit lesion in acute coronary syndrome patients with plaque erosion is linked to more susceptible plaque characteristics and a worse prognosis over time [42].

### 2.3 Plaque Rupture

Ruptured plaques often exhibit intimal ripping, disruption, or cap dissection and appear in IVOCT as regions of signal loss and/or irregular morphology (Fig. 1) [1]. A previous study revealed that patients with acute coronary syndrome presenting with plaque rupture as the culprit lesion on IVOCT had a worse prognosis than patients with intact fibrous caps did [42].

## 3. AI-based Methods for Characterizing Plaque Composition

This section reviews the most relevant AI models developed for plaque composition characterization, as summarized in Tables 1,2,3. It begins with approaches based on ML techniques, progresses to more recent DL applications, and concludes with examples of hybrid approaches that integrate both ML and DL elements.

### 3.1 Methods Based on ML

**Fig. 2.**
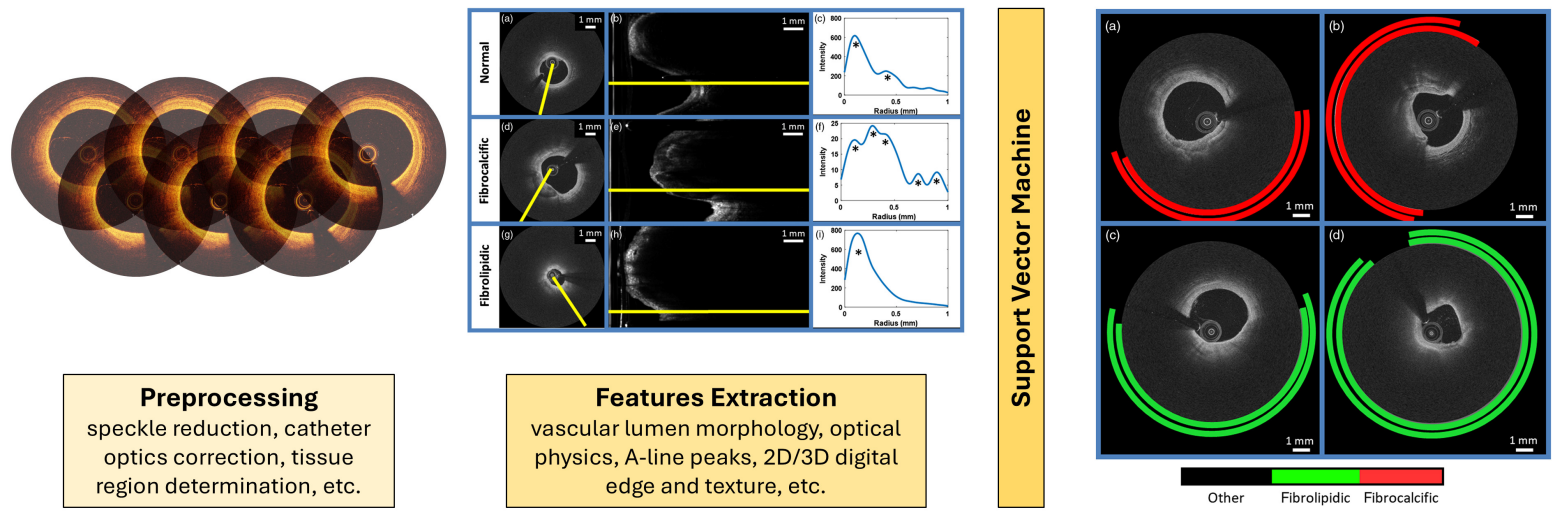
**Main pipeline of machine learning (ML)-based systems for plaque composition characterization from intravascular optical coherence tomography**. Specifically, the process outlined by Prabhu *et al*. [43], which includes the preprocessing phase, features extraction phase, ML model application, and A-line classification, is detailed as an example. Adapted with permission from [43]. 2D, 2-Dimensional; 3D, 3-Dimensional.

This section presents and compares the most relevant ML-based methods proposed in the literature over recent years for plaque composition characterization (Table 1, Ref. [43, 44, 45, 46, 47, 48, 49, 50, 51]). In general, each ML pipeline follows a similar structure, as illustrated in Fig. 2 (Ref. [43]), applying different models after the feature extraction step. The process begins with a preprocessing phase to clean, normalize, and correct the IVOCT images, preparing them as inputs for the downstream analysis. The next step is the feature extraction phase, where visual features are extracted in numerical format, containing potentially useful information for identifying plaque components. These data are then used as inputs to feed the ML algorithm and train it for the classification or segmentation task, which classifies and/or segments the images.

**Table 1.** **List of published studies that applied machine learning-based models to intravascular optical coherence tomography images for atherosclerotic plaque characterization, focusing on plaque composition, microstructure characterization, and plaque rupture identification**.

First author, year [Ref.]	Plaque characterization			Development dataset	AI architecture	Test performance	External validation	Consensus expert validation	Commercial software	Execution time
	Plaque composition	Plaque microstructure	Plaque rupture							
Athanasiou, 2011 [44]	Fibrous, calcified, lipidic, and mixed plaques image pixel classification	No	No	3 patients (50 frames)	Random forest classifier	Overall accuracy 0.80, 0.93 (fibrous), 0.82 (calcified), 0.77 (lipidic), 0.69 (mixed)	No	No	No	NA
Ughi, 2013 [45]	Fibrous, calcified, and lipidic plaques image pixel classification	No	No	49 patients (64 frames)	Random forest classifier	Overall accuracy 0.82, 0.90 (fibrous), 0.72 (calcified), 0.80 (lipidic)	No	No	No	30 s per frame
Athanasiou, 2013 [46]	Calcified plaques image pixel classification	No	No	10 patients (27 frames)	K-means algorithm	Sensitivity 0.83	No	Two experts independently examined the IVOCT images and detected all the calcified plaques	No	5 s per frame
Athanasiou, 2014 [47]	Fibrous, calcified, and lipidic plaques image pixel classification	No	No	22 patients (200 frames)	K-means algorithm (plaque segmentation), random forest classifier (image pixel classification)	Overlapping - nonoverlapping area, 0.87–0.37 (fibrous), 0.81–0.35 (calcified), 0.71–0.51 (lipidic)	No	2 experts selected 200 images and labeled them	No	40 s per frame
Rico-Jimenez, 2016 [48]	Fibrous, fibro-lipidic, and superficial-lipidic plaques A-line classification	No	No	57 cadaveric human coronary arteries, with histological samples for validation	Linear discriminant analysis classifier	Sensitivity 0.83 (fibrous), 0.85 (fibro-lipidic), 1.00 (superficial-lipidic)	Sensitivity 0.77 lipidic (visual), 0.86 (automatic)	Visual assessment performed by a trained IVOCT expert compared to the results obtained with the automatic approach	No	1.5 s per frame
Xu, 2016 [49]	No plaque, fibrous, fibrocalcified plaques, detect and fibroatheroma classification	No	Yes	47 patients (1000 frames)	Support vector machine classifier	Overall accuracy 0.90, 0.94 (no plaque), 0.95 (fibrous), 0.85 (fibrocalcified), 0.86 (fibroatheroma), 0.89 (plaque rupture)	No	No	No	NA
Zhou, 2017 [50]	Lipidic, and mixed plaques image pixel classification	No	No	9 patients (217 frames)	Random forest classifier	Accuracy 0.92 (lipidic), 0.78 (mixed)	No	No	No	10.9 s (computing features) and 1.7 s (classification) per frame
Huang, 2018 [51]	Calcified, lipidic, and fibrous plaques image pixel classification	No	No	11 patients (28 frames)	Support vector machine classifier	Overall accuracy 0.83, 0.79 (calcified), 0.87 (lipidic), 0.89 (fibrous)	No	3 well-trained IVOCT image readers implemented manual segmentation	No	2–3 min per frame
Prabhu, 2019 [43]	Fibrocalcified, fibro-lipidic, and mixed plaques A-line classification	No	No	*In vivo*: 49 pullbacks (6556 frames), *ex vivo*: 10 pullbacks (440 frames)	Support vector machine	Overall accuracy 0.82, sensitivity 0.75 (fibrocalcified), 0.94 (fibro-lipidic), 0.81 (other)	No	NA	No	3 s per frame (excluding features computation)

Note: AI, artificial intelligence; IVOCT, Intravascular optical coherence tomography; NA, not available.

In this context, the pioneering work published by Athanasiou *et al*. [44] in 2011 is the first example of a semi-automatic method based on ML to classify atherosclerotic plaque in IVOCT images into four possible types: calcium, lipid pool, fibrous tissue, and mixed plaque. The authors extracted features from grayscale IVOCT images and used them to train a random forest classifier [52] able to perform a pixel-level classification of the plaque types. The dataset used to train and test the method was small, consisting of 50 IVOCT annotated frames from three patients, which limited the generalizability of the algorithm. The extracted features included intensity-based (entropy and mean value [53]) and texture-based (co-occurrence matrix [54] and local binary patterns [55]) image characteristics. For each pixel in the plaque areas, features were extracted from an 11 × 11 pixel neighborhood. Of the 50 annotated IVOCT frames, 46 were used for training and testing, whereas four were reserved for qualitative application examples. A 10-fold cross-validation was applied to the 46 frames, resulting in an overall accuracy of 80%. However, these IVOCT frames are part of a sequence and are not truly independent, potentially affecting the validity of the results, introducing bias, and possibly overestimating model performance.

In 2013, Ughi *et al*. [45] proposed a more generalized algorithm for the semi-automated characterization of atherosclerotic plaques in IVOCT images. The authors employed a random forest classifier [52], a ML algorithm based on ensemble learning that builds multiple decision trees and combines their outputs to improve classification accuracy and control overfitting. The random forest classifier was developed using geometrical and texture features (spatial gray-level dependence matrices [56, 57]) and coupled them with the estimation of the attenuation coefficient µ_t_(d), calculated for each image pixel, indicating the extent to which the light intensity is reduced as it passes through the tissue [58]. The dataset included 49 patients, with a total of 64 plaques manually identified by a single medical expert, who visually selected only one IVOCT frame per plaque to avoid redundant information during training and testing. A balanced training set of 3100 pixels for each class was created. The ML model classified each image pixel into four classes: fibrotic plaque, calcified plaque, lipid plaque, and outliers. The resulting classification was visualized as a color-coded tissue map overlaid on the original IVOCT image. The overall classification accuracy was 81.5%.

After these two foundational works [44, 45], several studies have explored plaque composition characterization in IVOCT using ML techniques, aiming to overcome the limitations of earlier approaches (Table 1). In particular, the main limitation of these first two studies was their semi-automatic workflow, which required the manual identification of a region of interest before the actual application of the method. This process is time-consuming and prone to potential bias introduced from experts’ annotations.

An automated method for detecting calcified plaques in IVOCT images was proposed by Athanasiou *et al*. [46] to address the aforementioned challenges. The first step of the method involves detecting the penetration area in each IVOCT frame, defined as the distance between the lumen border and the maximum penetration depth of IVOCT from the lumen border itself (1.5 mm). Lumen border detection was performed automatically, using a customized method described in the same paper [46]. The k-means algorithm [59], an unsupervised ML technique that partitions data into *k* distinct clusters based on feature similarity, was then applied to classify each pixel within the penetration area into three clusters. Calcium is identified as the radial region between two distinct colors, corresponding to the area beneath the catheter artifact (located at the border of the penetration area). The dataset used for this study consisted of 10 patients, each independently examined by two experts, resulting in a total of 27 IVOCT frames selected. The proposed method achieved a sensitivity of 83%. Athanasiou *et al*. [47] improved the generalizability of their previously proposed plaque characterization method [44] by developing an automatic approach to detect four tissue types: calcium, lipid tissue, fibrous tissue, and mixed tissue. The algorithm implemented the following steps: lumen border detection and lumen border expansion of 1.5 mm in the radial direction based on the gravitational center of the lumen border, plaque region identification within the expanded area between the lumen border and the expanded border, and tissue characterization. The plaque region was identified using a k-means algorithm. For classification, calcium was first identified based on the k-means results, and then a set of texture and intensity-based features [53, 55, 60, 61] was extracted from the remaining regions and used in a random forest classifier [52] to classify lipid tissue, fibrous tissue, and mixed tissue.

Another notable study was conducted by Rico-Jimenez *et al*. [48], who introduced a tissue characterization method for IVOCT images that does not rely on estimating optical characteristics from the scans. Instead, this approach leverages the intrinsic morphological properties of the so-called “A-lines” in IVOCT scans. Specifically, each A-line is modelled as a linear combination of N depth profiles, which are classified based on their morphological traits to determine the exact tissue type (i.e., Intimal-Thickening, Fibrotic, Superficial-Lipid, and Fibrotic-Lipid) using a linear discriminant analysis algorithm. The feature set included 11 morphological features extracted from the IVOCT images. The dataset consisted of 57 IVOCT pullbacks, and the overall accuracy of the automatic method was 88%. More in detail, the sensitivity for fibrous tissue was 83%, that for fibro-lipidic tissue 85%, and that for superficial lipidic tissue was 100%. The main limitation of the proposed method was that blood was not considered as a class, potentially leading to blood artifacts influencing the results [62].

Xu *et al*. [49] were the first to automatically classify the IVOCT images into 5 classes, including not only normal tissue, fibrous plaque, fibroatheroma, and fibrocalcific plaque, but also plaque rupture. To achieve this goal, they extracted texture features from IVOCT images, including the Fisher vector [63], intensity, local binary patterns [64], histograms of oriented gradients [65], and bag-of-visual words [66], all of which are descriptors capturing the surface texture and details in the images and can be used by ML algorithms. Then, they used these texture features to train a linear support vector classifier [67], a supervised ML algorithm used for classification problems, achieving overall accuracies of 93.5%, 95.0%, 85.5%, 89.0%, and 84.5%, for normal tissue, fibrous plaque, fibroatheroma, plaque rupture, and fibrocalcific plaque, respectively. A similar approach, involving support vector machines coupled with feature extraction, was also applied in other studies to identify calcified, lipidic, and fibrous plaques [51] as well as fibro-calcified, fibro-lipidic, and mixed plaques [43]. By transforming raw data into a structured representation, feature extraction enabled the enhancement of the support vector machine’s efficiency in distinguishing between different plaque categories.

As highlighted in this overview, an arsenal of ML algorithms has been proposed over the years to characterize atherosclerotic plaques using IVOCT images, each algorithm presenting unique advantages and limitations. The foundational studies by Athanasiou *et al*. [44] and Ughi *et al*. [45] demonstrated that the effectiveness of random forest classifiers is hampered by a manual feature extraction phase that could introduce bias and limit scalability. Automated methods, such as those later proposed by Athanasiou *et al*. [46], improved reproducibility but still faced limitations when simple clustering techniques such as k-means were used. Rico-Jimenez *et al*. [48] introduced a novel methodology based on the morphological features of “A-lines”, which outperformed manual methods but struggled with real-world issues, such as blood artifacts. Xu *et al*. [49] advanced the field by incorporating texture features and support vector machines, but their generalizability was constrained by model complexity and small sample sizes. In conclusion, while ML-based approaches for plaque characterization have progressed, many challenges remain to be faced. The reliance on small datasets and feature engineering continues to hinder clinical application. The development of automated, generalized systems that use larger, more diverse datasets to increase resilience and eliminate expert bias is imperative in this field.

### 3.2 Methods Based on DL

**Fig. 3.**
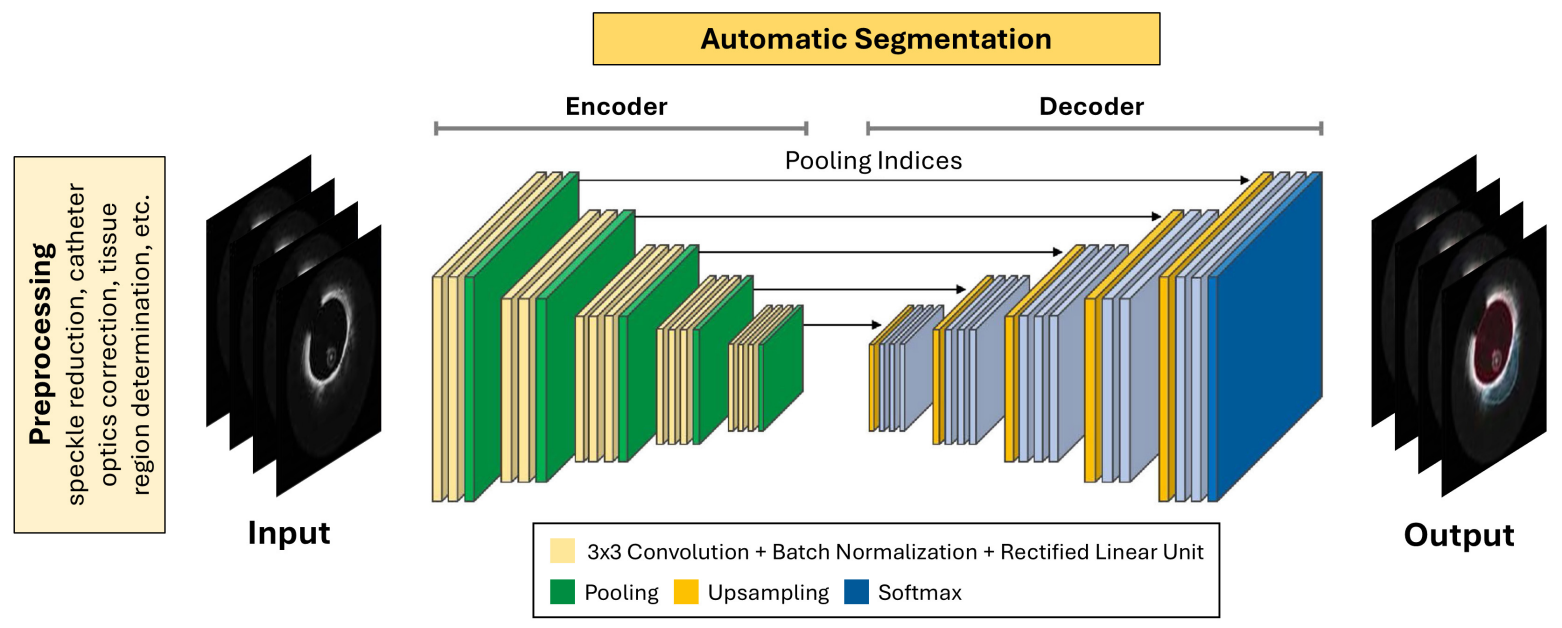
**Main pipeline of deep learning (DL)-based systems for plaque composition characterization from intravascular optical coherence tomography (IVOCT)**. The main steps involved in plaque composition characterization, including preprocessing phase, DL model application, and IVOCT image segmentation, are illustrated. Adapted with permission from [36] and [68].

This section presents and compares the most relevant DL-based methods proposed in the literature over recent years for plaque composition characterization (Table 2, Ref. [36, 68, 69, 70, 71, 72, 73, 74, 75, 76, 77, 78, 79, 80, 81, 82, 83, 84, 85, 86, 87, 88, 89, 90, 91]). In general, DL pipelines resemble ML pipelines, as illustrated in Fig. 3 (Ref. [36, 68]). In fact, as for ML pipelines, a preprocessing phase is first performed to clean, normalize, and correct the IVOCT images before they are used as input for the analysis. However, unlike ML pipelines, there is no explicit feature extraction phase in DL models, as these models directly extract features from images through their hidden layers. Compared with the ML approach, this process reduces errors or inaccuracies in feature extraction. Finally, the extracted features are used for image classification and/or segmentation purposes.

**Table 2.** **List of published studies that applied deep learning-based models to intravascular optical coherence tomography images for atherosclerotic plaque characterization, focusing on plaque composition, microstructure characterization, and plaque rupture identification**.

First author, year [Ref.]	Plaque characterization			Development dataset	AI architecture	Test performance	External validation	Consensus expert validation	Commercial software	Execution time
	Plaque composition	Plaque microstructure	Plaque rupture							
He, 2018 [69]	Calcified, lipidic, fibrous, and mixed plaques image pixel classification	No	No	22 patients (269 frames)	Convolutional neural network (CNN) architecture	Average sensitivity 0.87, 0.26 (calcified), 0.61 (lipidic), 0.90 (fibrous), 0.67 (mixed)	No	The ground truth data were manually established by expert observers	No	NA
Kolluru, 2018 [70]	Fibrocalcified, fibro-lipidic, and other plaques A-line classification	No	No	48 patients (4469 frames)	CNN architecture	Accuracy 0.78 (fibrocalcified), 0.87 (fibro-lipidic), 0.85 (other)	No	Consensus of 2 expert IVOCT readers	No	<1 s per frame
Gharaibeh, 2019 [71]	Calcified and other plaques segmentation	No	No	34 patients (2646 frames)	SegNet architecture	Dice coefficient 0.73 (calcified), 0.98 (other)	No	Consensus of 2 expert IVOCT readers	No	NA
Gessert, 2018 [72]	Plaques, calcified and lipidic/fibrous plaques classification	No	No	49 patients (4000 frames)	ResNet50-V2 and DenseNet-121 architectures	Accuracy 0.92 (plaque, ResNet50-V2), 0.78 (calcified), 0.85 (lipidic/fibrous), 0.90 (no plaque, DenseNet-121)	No	Consensus of 3 trained experts	No	NA
Athanasiou, 2019 [73]	Calcified, lipidic, fibrous, and mixed plaques image pixel classification	No	No	28 patients (700 frames)	CNN architecture	Accuracy 0.72 (calcified), 0.93 (lipidic), 0.96 (fibrous), 0.84 (mixed)	No	Consensus of 2 medical experts	No	NA
Gharaibeh, 2019 [68]	Calcified and other plaques segmentation	No	No	34 patients (2640 frames)	SegNet architecture	Dice coefficient 0.76 (calcified), 0.98 (other)	No	Consensus of 2 expert readers	No	NA
Lee, 2019 [74]	Calcified, lipidic, and other plaques A-line classification and segmentation	No	No	55 patients (4892 frames)	SegNet architecture	Dice coefficient 0.90 (calcified), 0.83 (lipidic) for A-lines classification, 0.73 (calcified), 0.80 (lipidic), 0.91 (other) for segmentation	600 frames. Dice coefficient 0.99 (other)	No	No	0.27 s per frame
Liu, 2020 [75]	Fibrous plaque detection	No	No	1000 frames	Multi-scale CNN	Accuracy 0.94	No	3 trained experts	No	NA
Abdolmanafi, 2020 [76]	Calcified and fibrous plaques classification	Macrophage and neovascularization classification	No	19 pullbacks	AlexNet, VGG-19, and Inceptionv3 architectures for feature extraction, random forest for classification	Accuracy 0.90 (calcified), 0.94 (fibrous), 0.92 (macrophage), 0.95 (neovascularization)	No	Each annotated image was reviewed by two cardiologists and if there was any disagreement, a consensus was reached by reviewing carefully each region of interest	No	NA
Baruah, 2020 [77]	Calcified, lipidic, fibrous, fibrocalcified, and fibroatheroma plaques segmentation	Thick versus thin-cap fibroatheroma segmentation	No	76 *ex vivo* coronary arteries (400 frames), 13 *in vivo* patients	Neural networks	Sensitivity 0.85 (calcified), 0.86 (lipidic), 0.87 (fibrous), 0.72 (fibrocalcified), 0.72 (fibroatheroma), 0.99 (thick-cap versus thin-cap fibroatheroma)	Sensitivity 0.80 (calcified), 0.82 (lipidic), 0.86 (fibrous)	2 expert IVOCT readers and 2 cardiovascular pathologists with guidance from histology	No	NA
Lee, 2020 [36]	Calcified plaques segmentation	No	No	68 patients (8231 frames)	3D CNN architecture (classification), SegNet: VGG-16 architecture (segmentation)	F1 score 0.92 (calcium classification), F1 score 0.78 (calcium segmentation)	Qualitative evaluation on 4320 *ex vivo* cadaveric images from 4 hearts with 4 vessels	Clinical images labeled by 2 expert cardiologists	No	0.3 s per frame
Abdolmanafi, 2021 [78]	Atherosclerotic tissue segmentation, fibrous, fibrocalcified tissue, and fibroatheroma classification	Micro-vessels, and thrombi tissue classification	No	41 pullbacks (2052 frames)	ResNet-based spatial pyramid pooling module, custom ResNet architecture coupled with sparse auto-encoder neural network architecture	BF-Score 0.84 (atherosclerotic tissue), 0.99 (fibrocalcified), 0.99 (fibroatheroma), 0.90 (micro-vessels), 0.98 (thrombi)	No	Trained operator with review by 2 cardiologists to reach a consensus	No	0.3 s per frame
Yin, 2021 [79]	Fibrous, calcified, and lipidic plaques image pixel classification	No	No	31 patients (2000 frames)	TwoPathCNN architecture	F1 score 0.86 (fibrous, calcified and lipidic average)	No	3 experienced observers manually segmented each image, double-checking the results. Disagreement was confirmed by an interventional cardiologist	No	2 s per frame
Avital, 2021 [80]	Calcified plaques segmentation	No	No	540 frames	U-Net architecture	Dice coefficient 0.71	No	Manual segmentation from 2 independent individuals	No	NA
Chu, 2021 [90]	Fibrous, calcified, and lipidic plaques segmentation	Cholesterol crystals, macrophages, and micro-vessels plaques segmentation	No	391 patients (11,673 frames)	U-shaped encoder-decoder architecture	Dice coefficient 0.91 (fibrous), 0.85 (calcified), 0.77 (lipidic), 0.53 (cholesterol crystals), 0.49 (macrophages), 0.60 (micro-vessels)	30 patients (300 frames). Accuracy 97.6% (fibrous), 90.5% (lipidic), 88.5% (calcified), 48.1% (macrophages), 94.7% (cholesterol crystals), based on consensus	Development: 2 experienced IVOCT analysts, verification by IVOCT specialist under supervision of senior cardiologist. External validation: 3 independent core labs. Consensus defined as agreement between ≥2 core labs	Integrated into OctPlus software (Pulse Medical Imaging Technology, Shanghai, China)	Median analysis time: 21.4 s per pullback
Cheimariotis, 2021 [81]	Plaques, fibrous, calcified, lipidic, and other plaques A-line classification	No	No	33 patients (183 frames)	CNN architecture	Accuracy 0.75 (plaques), overall accuracy 0.53 (fibrous, calcified, lipidic, and other), 0.83 (fibrocalcified and fibro-lipidic)	No	Expert cardiologists	No	NA
Rico-Jimenez, 2022 [82]	Lipidic plaques A-line classification	No	No	5 cadaveric human hearts (98 frames)	CNN-time-series classifier	Accuracy 0.90	No	No	No	9.6 ms per frame
Lee, 2022 [83]	No	Microchannel segmentation	No	41 patients (3075 frames)	Encoder (Xception network) decoder architecture coupled with CNN	Dice coefficient 0.81	No	Two expert cardiologists	No	NA
Shi, 2023 [84]	No	Thin-cap fibroatheroma A-line classification	No	2300 frames	Weakly supervised object detection method based on a deep CNN architecture	Sensitivity 0.88	No	Medical experts	No	0.51 s per frame
Lee, 2023 [91]	Plaque segmentation, calcified plaques segmentation	No	No	68 patients (8231 frames for calcified plaques classification, 8231 clinical, and 4320 *ex vivo* cadaveric frames for calcified plaques segmentation)	3D CNN model (classification), SegNet (segmentation)	Dice coefficient 0.92 (calcified classification), 0.78 (calcified segmentation)	34 pullbacks (2723 frames), 12.2% of the frames required significant manual modifications	No	OCTOPUS software 2023.3, (based on Matlab R2019b, MathWorks, Natick, MA, USA)	0.6 s per frame
Tang, 2023 [85]	Calcified and lipidic plaques segmentation	No	No	14 patients (2388 frames, 378 with calcium and 1355 with lipid)	Self-attention ResNet (classification), Convolution AutoEncoder (CAE) U-Net (segmentation)	Accuracy 1.00 (calcified), 0.98 (lipidic), Dice 0.72 (calcified), 0.60 (lipidic)	No	Labels delineated by experienced cardiologists	No	NA
Wang, 2023 [86]	No	Macrophages, cavities/dissections, and thrombi classification	No	69 patients (2988 frames)	G-Swin transformer architecture	Sensitivity 0.91 (macrophages), 0.90 (cavities/dissections), 0.95 (thrombi)	No	2 expert physicians, senior doctors	No	NA
Liu, 2024 [87]	Calcified plaques segmentation	No	No	62 clinical pullbacks (2151 frames)	Transformer-based pyramid network	Dice coefficient 0.84	No	Yes	No	74.20 ms per frame
Lee, 2024 [88]	No	Fibrous cap segmentation	No	77 patients (8970 frames)	Modified SegNet architecture	Dice coefficient 0.85	74 patients (1362 frames). Dice coefficient 0.85	OCTOPUS software and 2 experts	No	0.02 s per frame
Chu, 2024 [89]	No	Thrombi segmentation	No	339 patients (5649 frames)	Multi-head crossattention, self-attention, and convolutional-based feedforward network	Dice coefficient 71%	52 patients (548 frames)	Two experienced IVOCT analysts	No	2–3 s per pullback

Note: VGG, visual geometry group; BF-Score, Boundary F1 Score.

In this context, the first studies employing DL methods for characterizing plaque composition date back to 2018 [69, 70]. In particular, He *et al*. [69], proposed an approach based on a CNN for the automatic identification of plaque components in IVOCT images. The five identified classes were lipid tissue, fibrous tissue, mixed tissue, calcified tissue, and background. The method consisted of two steps: extracting the IVOCT tissue area using Otsu’s automatic thresholding [92], and performing a CNN-based pixel classification for each image patch. The dataset used in the study included 269 IVOCT images acquired from 22 patients. The average prediction accuracy of the CNN-based approach was 87%, with sensitivity values of 97%, 61%, 90%, 67%, and 26% for background, lipid tissue, fibrous tissue, mixed tissue, and calcified tissue, respectively. The results indicated low performance in the characterization of calcium. This might be due to the low number of pixels labeled as calcium. Kolluru *et al*. [70] also applied a CNN-based approach to classify IVOCT A-lines into fibrocalcific (i.e., fibrous layer followed by calcification), fibrolipid (i.e., fibrous layer followed by lipid deposit), or other layers. Their dataset comprised 48 patients and 4469 IVOCT frames, labeled by consensus of two expert image readers. The model performance was assessed using 10-fold cross-validation using held-out data, increasing the robustness of the proposed method. They achieved accuracies of 77.7 ± 4.1%, 86.5 ± 2.3%, and 85.3 ± 2.5% for fibrocalcific, fibrolipid, and other layers, respectively.

Other examples of standard CNN architecture applications for plaque composition characterization are represented by the studies by Athanasiou *et al*. [73] and Cheimariotis *et al*. [81]. In the former study [73], the goal was to subdivide the arterial wall into six classes: calcium, lipid tissue, fibrous tissue, mixed tissue, non-pathological tissue, and no visible tissue. The dataset contained 700 IVOCT frames from 28 patients, annotated by two medical experts. The reported accuracies were 72%, 93%, 96%, 84%, and 99% for calcium, lipid tissue, fibrous tissue, mixed tissue, and non-pathological tissue, respectively. Calcium remained the most challenging class to predict, although improvements have been observed compared to the study of He *et al*. [69]. In the latter study [81], the aim was to automatically classify IVOCT image A-lines into four classes: lipid, mixed, fibrous, and calcified plaque. The dataset was made of 183 frames from 33 patients. The overall accuracy was 53%, whereas when the classification of fibrocalcific versus fibrolipid plaques was considered, it was 83%.

Other groups have proposed slightly different versions of the standard CNN architecture. Liu *et al*. [75] introduced a multi-scale CNN consisting of a contracting path and an extracting path to generate a bounding box identifying fibrous plaques, achieving an accuracy of 94%. Lee *et al*. [36] applied a three-dimensional CNN to classify calcified plaques, reporting a sensitivity of 97.7 ± 2.4% and an F1 score of 92.2 ± 2.1%. Yin *et al*. [79] proposed a two-path CNN architecture to classify each IVOCT image pixel into fibrous, calcified, or lipidic categories, achieving sensitivities of 95 ± 3%, 87 ± 6%, and 81 ± 11% for fibrous, calcified, and lipidic classes, respectively.

Several studies have employed U-Net architectures for plaque segmentation tasks in IVOCT [93]. These architectures are characterized by a symmetric encoder-decoder structure with skip connections that preserve spatial information. Three relevant examples of U-Net applications are those of Avital *et al*. [80], Tang *et al*. [85], and Chu *et al*. [90]. Avital e*t al*. [80] used a U-Net model to segment calcified plaques, resulting in a Dice coefficient of 71%, while Tang *et al*. [85] used a U-Net architecture to segment calcified and lipid plaques, obtaining Dice coefficients of 72% and 60%. Chu *et al*. [90] also used a U-shaped encoder-decoder architecture based on U-Net principles to segment fibrous, calcified, and lipidic plaques. Their model achieved Dice values of 91% for fibrous plaques, 85% for calcified plaques, and 77% for lipid plaques. External validation of this study revealed good plaque classification accuracy, with 97.6% for fibrous, 88.5% for calcified, and 90.5% for lipidic plaques, as validated by three separate core labs. The algorithm was integrated into the OctPlus software (Pulse Medical Technology Inc., Shanghai, China), enabling practical implementation and real-time plaque analysis. The features of this software are detailed in Section 6.

An improvement over the standard CNN-based approach involves leveraging pre-trained neural networks through transfer learning. Popular networks employed in these techniques are AlexNet [94, 95], Visual Geometry Group (VGG) models [95, 96], and GoogleNet [97]. AlexNet, one of the first models to demonstrate the power of DL in image classification, uses convolutional layers to extract hierarchical features. The VGG models, known for their simplicity and depth, focus on using small convolutional filters while increasing the network depth, making them particularly effective for detailed feature extraction. GoogleNet, with its innovative Inception modules, introduced the concept of multi-scale feature extraction, allowing for more efficient and flexible architectures. Among the variations of GoogleNet, the Inception-v3 model [95, 97, 98] refines these concepts, offering improvements in accuracy and computational efficiency through techniques like factorized convolutions and label-smoothing. In this context, Gessert *et al*. [72] applied transfer learning to detect the presence or absence of atherosclerotic plaques in IVOCT images and further classified plaque types as calcified or fibrous/lipid. Their dataset included 4000 IVOCT frames from 49 patients. The authors used ResNet50-V2 [99] and DenseNet-121 [100] architectures, considering both Cartesian and polar representations of IVOCT images as input. ResNet50-V2 leverages residual connections to ease the training of deeper networks, while DenseNet-121 employs dense layer connections for efficient feature reuse. The best results were obtained using ResNet coupled with the Cartesian representation of IVOCT images, which achieved 90% accuracy for plaque classification. The class-specific accuracies were 78% for calcified plaques, 85% for fibrous/lipid plaques, and 90% for the absence of plaques. Abdolmanafi *et al*. [78] applied transfer learning to perform semantic segmentation of atherosclerotic tissues, followed by a sparse-autoencoder, designed to enforce sparsity in neural activations, for tissue characterization into fibrous or fibrocalcified plaques, fibroatheroma, thrombus or microvessel. In the first step, a ResNet-based spatial pyramid pooling module was employed. In the second step, sparse-autoencoders were trained on the CNN features extracted during the segmentation phase, with a softmax layer used for tissue classification. This approach achieved a Boundary F1 Score (BF-Score) of 84 ± 18% for semantic segmentation and classification accuracies of 96%, 99%, and 99% for fibrous plaques, fibrocalcific plaques, and fibroatheromas, respectively. The results for thrombi and microvessels are presented in Section 4.

In the context of transfer learning approaches, other studies have utilized the SegNet architecture [101], an encoder-decoder network optimized for semantic pixel-wise segmentation. In particular, IVOCT image calcium segmentation works were presented by Gharaibeh *et al*. in 2019 [68, 71]. The authors employed a pre-trained CNN architecture for semantic pixel-wise segmentation SegNet [101], to segment calcifications inside IVOCT images. Their dataset comprised 2640 IVOCT frames from 34 patients, labeled through consensus between two expert readers. The achieved Dice coefficients were 76 ± 3%, 98 ± 1%, and 98 ± 1% for calcifications, lumen, and other tissue segmentation, respectively. An additional application of a pre-trained SegNet architecture was that by Lee *et al*. [74], who segmented both lipidic and calcified plaques, obtaining a Dice coefficient of 80 ± 4%, 73 ± 9%, 91 ± 3% for fibrolipid, fibrocalcific, and other tissue segmentation. More recently, Lee *et al*. [91] developed the “Optical Coherence TOmography PlaqUe and Stent (OCTOPUS)” analysis software, which employs the SegNet architecture (pre-trained) for segmenting calcified plaques. Further details about this software are provided in Section 6. The approaches presented in this section, which are rapidly being integrated into clinical software, provide real-time analysis and increased plaque categorization accuracy, improving the objectivity of IVOCT-guided treatments.

### 3.3 Hybrid-based Systems

As described in Sections 3.1 and 3.2, ML-based and DL-based approaches are increasingly used in plaque composition characterization in IVOCT images, leveraging their ability to automatically discover patterns and characteristics from large datasets. These methods often rely on conventional ML classifiers, such as support vector machines and random forests, or fully on DL models, such as CNNs or transfer learning approaches. While effective, these stand-alone techniques may struggle to capture the complexities of plaque heterogeneity, especially in cases where limited datasets are available for model training and validation, or where complex anatomical structures are involved.

In contrast, hybrid ML approaches combine the capabilities of both traditional ML and DL techniques, offering greater flexibility, interpretability, and generalizability abilities (Table 3, Ref. [102, 103, 104]). They exploit the advantages of deep feature extraction while avoiding the hazards of overfitting inherent in purely DL models. For example, Xu *et al*. [102] achieved an accuracy of 76% by combining deep features extracted from the VGG-19 network [78], a deep CNN known for its depth and feature extraction capabilities [79], with a linear support vector machine [67] for fibroatheroma classification. This hybrid model effectively leveraged the powerful representational capabilities of CNNs while avoiding overfitting in smaller datasets by integrating deep feature extraction with a simpler classifier. Abdolmanafi *et al*. [103] designed a tissue characterization model to detect pathological formations (i.e., calcifications, fibrosis, macrophages, neovascularization), alongside normal coronary artery tissues, specifically caused by Kawasaki disease [105, 106]. Although its clinical application is highly specific, as it is related to a very specific disease, the study is particularly noteworthy for the proposed methodology and for the characterization of atherosclerotic plaques. The dataset consisted of 33 patients (3149 IVOCT frames). The output classes included calcification, fibrosis, macrophages, and neovascularization. The CNNs AlexNet, VGG-19, and Inception-v3 were used as feature extractors, combined with a random forest classifier as the final classifier applied to the extracted features. AlexNet, VGG-19, and Inception-v3 are all CNNs designed for image analysis. Specifically, AlexNet, one of the first successful CNNs, uses multiple convolutional layers with rectified linear unit (ReLU) activation function to extract features [95]. VGG-19 employs a deeper architecture with 3 × 3 convolutions to enhance hierarchical feature learning [95]. Inception-v3 introduces inception modules, which combine convolutions of different sizes to capture patterns at multiple scales [95]. The best-performing architecture was a random forest with majority voting, which incorporated all three CNN models as feature extractors. The model achieved accuracies of 95 ± 5% and 95 ± 4% for calcification and fibrosis, respectively. The results for macrophages and neovascularization are reported in Section 4, which focuses on plaque microstructure characterization. Similarly, Lee *et al*. [104] applied a hybrid technique to classify fibrocalcific plaques, fibro-lipidic plaques, and other plaque types by integrating deep convolutional features with hand-crafted lumen morphological parameters. The two above-mentioned sets of features were used to train and evaluate a random forest classifier, which produced high sensitivity outputs (91.2 ± 6.4% for fibrocalcific plaques, 84.8 ± 8.2% for fibro-lipidic plaques, and 96.5 ± 1.1% for other plaques), and F1 scores (71.9 ± 7.0% for fibrocalcified plaques, 88.7 ± 4.0% for fibro-lipidic plaques, and 97.7 ± 0.8% for other plaques). The integration of DL’s ability to capture complicated, high-level characteristics with hand-crafted features emphasizing domain-specific anatomical elements proved particularly beneficial.

**Table 3.** **List of published studies that applied hybrid artificial intelligence-based approaches to intravascular optical coherence tomography images for atherosclerotic plaque characterization, focusing on plaque composition, microstructure characterization, and plaque rupture identification**.

First author, year [Ref.]	Plaque characterization			Development dataset	AI architecture	Test performance	External validation	Consensus expert validation	Commercial software	Execution time
	Plaque composition	Plaque microstructure	Plaque rupture							
Xu, 2017 [102]	Fibroatheroma classification	No	No	18 patients (360 frames)	Deep features extracted by VGG-19 coupled to linear support vector machine classifier	Accuracy 0.76	No	Trained experts labeled the frames	No	NA
Abdolmanafi, 2018 [103]	Calcified and fibrous plaques classification	Macrophage and neovascularization classification	No	33 patients (3149 frames)	AlexNet, VGG-19, and Inceptionv3 architectures for feature extraction, random forest for classification	Accuracy 0.95 (calcified), 0.95 (fibrous), 0.91 (macrophage), 0.98 (neovascularization)	No	Annotated images are validated by expert cardiologists	No	NA
Lee, 2020 [104]	Fibrocalcified, fibro-lipidic, and other plaques A-line classification	No	No	49 patients (6556 frames)	Hybrid learning approach, combining deep learning convolutional and hand-crafted, lumen morphological features. Random forest classifier	F1 score 0.72 (fibrocalcified), 0.89 (fibro-lipidic), 0.98 (other)	No	Labels delineated by 2 experienced readers	No	1 s per frame

## 4. AI-based Methods for Characterizing Plaque Microstructure

This section presents and compares the most relevant DL-based methods proposed in the literature over recent years for plaque microstructure characterization (Table 2). To the authors’ knowledge, no studies have focused solely on ML-based approaches for plaque microstructure characterization, unlike plaque composition characterization (Section 3 of this review).

Concerning TCFA identification, Baruah *et al*. [77] developed a system that, in addition to categorizing plaque types (i.e., calcified, lipidic, and fibrous), differentiated between thick- and TCFAs using neural networks. Their dataset included pullbacks from 76 *ex vivo* coronary arteries (400 IVOCT frames) and 13 patients. They obtained accuracy scores of 72% for fibroatheroma detection and 99% for distinguishing thin-cap versus thick-cap fibroatheromas, based on comparison with s graded against the pathologists’ classification. The patients were used for a qualitative performance analysis. Shi *et al*. [84] introduced a weakly supervised object detection approach that uses a deep CNN architecture to classify TCFA, reporting a sensitivity of 88%. Although expert readers verified this approach, it lacked external validation and commercial implementation. In these studies [77, 84], the TCFA was identified, but no estimate of the fibroatheroma thickness or its location within the IVOCT frame was provided. More recently, Lee *et al*. [88] employed a modified SegNet architecture to segment fibrous caps, allowing for the estimation of fibroatheroma thickness. Their system was applied to both development (8970 IVOCT frames) and external (1362 IVOCT frames) datasets, analyzing a total of 77 patients. The model achieved a Dice coefficient of 85% in both cases. This approach was also integrated into the OCTOPUS software (see Section 6 for more details on this software).

Concerning the presence of structures such as cholesterol, macrophages, and micro-vessels, as anticipated in Section 3.3, Abdolmanafi *et al*. [103] employed AlexNet, VGG-19, and Inceptionv3 CNN architectures for feature extraction, combined with a random forest classifier, to classify macrophages and neovascularization, among other features. Using a dataset of 3149 IVOCT frames from 33 patients and considering annotations from expert cardiologists in model training, they achieved remarkable accuracy (91 ± 6% for detecting macrophages and 98 ± 2% for neovascularization). However, despite its high performance, the lack of external validation and commercial implementation restricts its broader clinical applicability. In subsequent work, Abdolmanafi *et al*. [78] developed a ResNet-based spatial pyramid pooling module combined with sparse auto-encoders to classify micro-vessels, achieving an accuracy of 90% as validated by expert IVOCT readers. Chu *et al*. [90] utilized a U-shaped encoder-decoder architecture to segment cholesterol crystals, macrophages, and micro-vessels, among other IVOCT features. This model, validated internally on 391 patients and externally on 30 patients, achieved Dice coefficients of 53%, 49%, and 60% for these features, respectively. Importantly, this model was integrated into the commercial OctPlus software, enhancing its clinical applicability (see Section 6 for details on this software). Lee *et al*. [83] applied an encoder-decoder architecture based on the Xception network for microchannel segmentation, achieving a Dice coefficient of 81%. In the last two studies [83, 90], thanks to the segmentation approach, a measure of the location and the area of the plaque components is also given. Finally, Wang *et al*. [86] used the G-Swin transformer [107], a vision transformer architecture that uses hierarchical representations and shifted windows for efficient feature extraction, to classify macrophages and cavities/dissections, achieving sensitivities of 91% and 90%, respectively.

Concerning thrombus identification, Abdolmanafi *et al*. [78] developed a ResNet-based spatial pyramid pooling module that works with sparse auto-encoders, which can also classify thrombi. The method, validated by expert IVOCT readers, achieved an accuracy of 98% for thrombi. Wang *et al*. [86], as previously mentioned, employed the G-Swin transformer [107] for thrombus classification, achieving a sensitivity of 95%. Finally, Chu *et al*. [89] introduced the first automatic thrombus segmentation in IVOCT using a dual-coordinate cross-attention transformer network, which can be readily integrated into other established transform models to improve performance. Unlike previous studies [78, 86], Chu *et al*. [89] also provides an estimate of thrombus location and area. Their model, trained on 5649 IVOCT frames from 339 patients, was externally tested on 548 frames from 52 individuals, achieving a Dice coefficient of 71%.

## 5. AI-based Methods for Detecting Plaque Rupture

To the authors’ knowledge, the work of Xu *et al*. [49] (Table 1) is the only study that has applied AI-based methods to IVOCT images for detecting the presence of plaque rupture. More in detail, a support vector machine classifier, using image texture features, was employed to classify IVOCT images into five classes: normal, fibrous plaque, fibroatheroma, plaque rupture, and fibrocalcified plaque. The dataset consisted of 1000 IVOCT frames from 47 patients, annotated manually by one single cardiologist. The mean accuracy for plaque rupture detection was 89%. The main limitation of this study is represented by the manual labels provided by only one cardiologist (no consensus was employed). This work opens the way to testing more advanced AI-based methods (such as DL approaches) to perform plaque rupture detection.

## 6. AI-based Software

While numerous AI approaches have been developed for assessing coronary artery plaques in IVOCT in research, they are not yet widely available for clinical usage. In general, published studies lack access to source code, data, and trained models in public repositories, hindering reproducibility and broader implementation. The only available software for plaque assessment from IVOCT are the OCTPlus software (Pulse Medical Imaging Technology, Shanghai, China) [90], the OCTOPUS software [91], and the Ultreon software (Abbott Laboratories, Abbott Park, IL, USA) [108]. Among these, Ultreon is the only software to have received both FDA and CE approval (FDA 510(k), CE mark April 2021, approval number: K210458) [109, 110].

OCTPlus [90], whose AI-based method was introduced in Section 3.2, is intended for the automated analysis of IVOCT pullbacks. The software accurately segments and characterizes atherosclerotic plaques, identifying components such as fibrous and lipidic tissue and calcifications. The OCTPlus interface displays both 2-Dimensional (2D) cross-sectional and 3-Dimensional (3D) views of the analyzed regions, offering an intuitive representation of plaque composition. Users can quantify plaques, perform real-time analysis, and detect markers of plaque instability, including macrophages, cholesterol crystals, and microvessels.

OCTOPUS [91], whose AI-based method was introduced in Section 3.2, offers automated analysis of IVOCT pullbacks, enabling both plaque and stent evaluation. The software provides lumen and calcification segmentation, and assesses stent deployment by recognizing struts, categorizing them as covered or uncovered, and quantifying tissue coverage and malapposition.

**Fig. 4.**
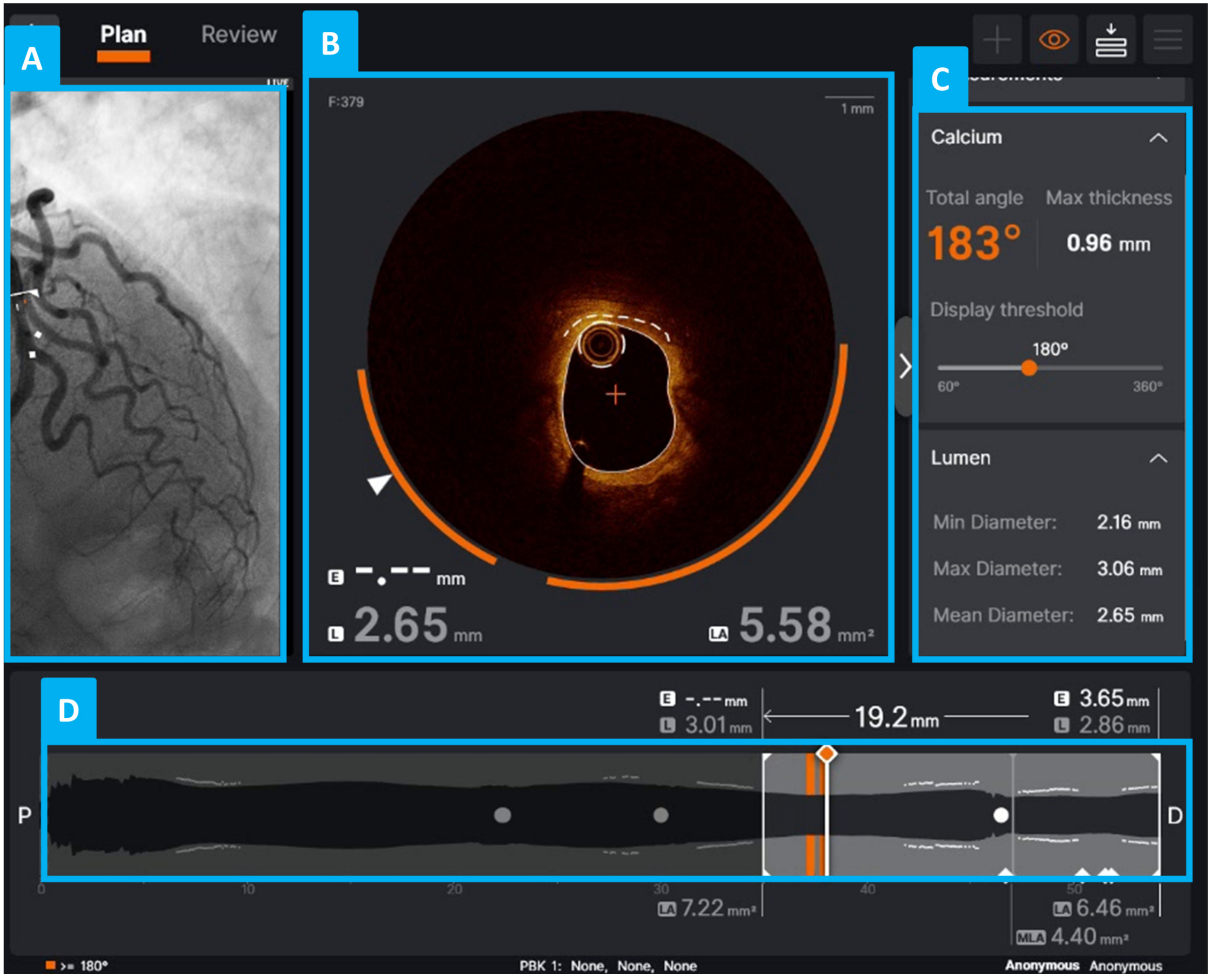
**Graphical user interface of the Ultreon 2.0 software (Abbott Laboratories)**. (A) Angiographic view of a coronary vessel imaged using IVOCT. (B) Example of a cross-sectional IVOCT image (i.e., IVOCT frame) of the analyzed vessel, with Ultreon’s morphological assessment view showing automated calcium detection (calcium is highlighted by an orange arc). (C) Quantitative display of the calcium arc, thickness, and longitudinal extent, along with lumen diameter information. (D) Longitudinal reconstruction of the vessel from IVOCT imaging data: the current IVOCT frame is indicated by a thicker white line, while IVOCT frames with a total calcium angle greater than 180° are marked by orange lines.

Ultreon [108] automatically detects the lumen, stent, external elastic membrane, and calcium (Fig. 4) [109, 110]. One of its most notable features for atherosclerotic plaque characterization is its advanced calcium detection and analysis. Using AI-based algorithms, the software automatically identifies calcified plaques on IVOCT frames and provides measurements of the calcium arc and thickness.

## 7. Challenges and Limitations of the Current AI Approaches

AI-based approaches for studying atherosclerosis and characterizing plaque formations in coronary artery images, particularly IVOCT images, have advanced extraordinarily in recent years [11]. However, many open issues remain to be addressed. In general, the application of AI in detecting atherosclerotic disease remains a “black-box” concept that hampers the implementation of AI-based tools in clinical processes [111]. Although AI technologies have become more common in simple segmentation tasks, AI-based models for risk prediction are still approached with caution [112], primarily due to a lack of explainability and interpretability. To address this perception, several factors should be considered.

The datasets used in computational studies should be carefully evaluated for both quantity and quality. Specifically, it is crucial to conduct large-scale multicenter studies to assess the AI-based models in an unbiased, reproducible, and statistically significant manner. External validation datasets are necessary to confirm the prognostic power of the developed methodologies. Moreover, benchmarking datasets are urgently needed to enable fair testing and comparisons of the various models developed over the years. Additionally, ensuring the robustness of ground truth labels and segmentations performed by human operators is crucial to minimize bias caused by inter- and intra-observer variability. One potential solution to reduce this bias is the use of histopathological data from post-mortem studies, as seen in a few studies [43, 48, 77, 82, 91].

Another critical issue is the risk of shortcut learning in DL models, where the algorithm may memorize spurious correlations or irrelevant patterns present in the training data rather than learning true disease-related features. This issue is particularly relevant in medical imaging, where biases in the dataset, such as artifacts or acquisition-specific features, can mislead the learning process. To mitigate this, it is important to carefully select model architectures that are appropriate for the size and diversity of the available data and to apply rigorous data augmentation strategies during training to improve generalizability [113].

Furthermore, integrating AI models into healthcare software and workflows would markedly increase their practical use. Clinicians need models that are not only accurate in their predictions, but also interpretable, explainable, and easy to integrate into existing hospital information systems and electronic health records. The development of user-friendly interfaces and visualization tools is essential to support AI-driven decision-making. However, regulatory approval and compliance with clinical guidelines must be addressed before these models can be fully implemented in healthcare settings. This is a critical aspect, considering that due to strict regulations bringing novel medical devices or software to market is a complex and time-consuming procedure. Multiple stages are needed, including pre-clinical studies, clinical trials, and comprehensive documentation to demonstrate safety, efficacy, and industry compliance. Regulatory agencies often mandate extensive testing and review, which can take several years. Additionally, ongoing post-market surveillance and the need to comply with evolving regulations further complicate the process from innovation to commercialization, making it both time-consuming and resource-intensive. Finally, continuous updates to the models and real-time learning from new patient data will be essential to ensure that AI tools remain relevant and perform effectively in clinical practice.

## 8. Toward Integration Into Clinical Risk Prediction Systems

**Fig. 5.**
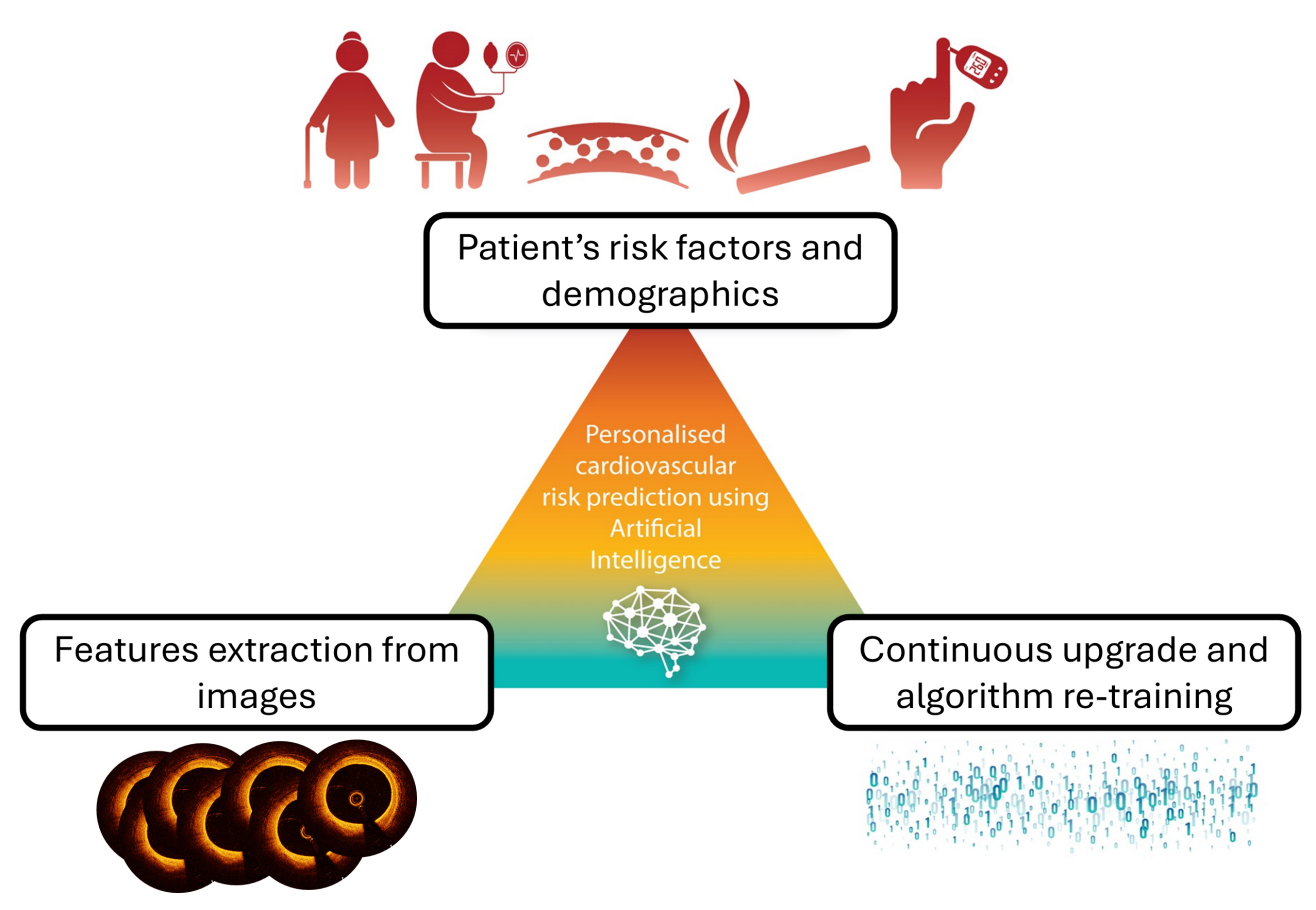
**Schematic of the process for integrating image-derived biomarkers with patients’ clinical characteristics in artificial intelligence models to evaluate coronary artery disease, assess risk, and create individualized medical care**. Adapted with permission from [114].

AI-based algorithms can not only automate the classification and segmentation of the lumen contours, stent struts, plaque components and other features in IVOCT images, but also integrate imaging biomarkers with established cardiovascular risk factors to improve the prediction of event recurrence at follow-up (Fig. 5, Ref. [114]), as demonstrated with other imaging techniques. For example, in coronary computed tomography angiography (CCTA), the standardized fat attenuation index (FAI) score is used in a risk prediction algorithm alongside variables such as age, gender, and cardiovascular risk factors such as hypertension, diabetes, smoking, and hyperlipidemia, to calculate the absolute 8-year risk of fatal cardiac events [114]. Integrating image-derived biomarkers, particularly those extracted from IVOCT alone [11] or in combination with other imaging modalities such as CCTA [115], with established cardiovascular risk factors in AI-based models for predicting adverse events at follow-up could provide additional prognostic insights for patients with coronary artery disease and help predict the risk of death, new myocardial infarction, or coronary artery stenosis. This approach may lead to more accurate risk prediction in symptomatic patients undergoing coronary angiogram than traditional risk scores, which are based solely on clinical factors and were originally designed for primary prevention in the general population. Another possibility is the use of biomarkers extracted from IVOCT images to build cardiovascular risk predictors. While this is a challenging task, to the best of the authors’ knowledge, no study has yet successfully addressed this issue using IVOCT. A study by Araki *et al*. [116] addressed a similar problem by employing ML techniques to predict coronary artery disease based on plaque morphology information extracted from IVUS, but a major adverse cardiovascular risk predictor system based on IVOCT data is still lacking. Establishing large-scale multinational cohorts could further pave the way for developing novel imaging biomarkers linked to reliable outcomes, advancing the creation of clinical risk prediction systems in secondary prevention. By incorporating adverse events over an extended follow-up period, AI-based models could be trained to predict atherosclerotic plaque vulnerability and assess patient risk. This approach would enable a more comprehensive risk evaluation, identifying high-risk individuals and guiding personalized treatment plans that target specific risk factors.

## 9. Conclusions

This work provides a comprehensive overview of AI-based approaches for identifying atherosclerotic plaque components in IVOCT images of coronary arteries. Despite significant advances in this research field, only a few systems are commercially accessible and used in clinical settings. The analysis highlights key limitations, such as a lack of standardization, reproducibility, explainability, interpretability, and integration into existing clinical systems, which are critical factors for developing effective AI-based models. Addressing these gaps is essential, particularly for atherosclerotic plaque characterization and adverse event risk prediction. While advanced methods have improved over time, there is an urgent need for standardized applications to assess major adverse cardiovascular events in clinical practice, as well as regulatory approval. With additional testing using large-scale outcome cohorts, these models can be recalibrated and updated regularly, eventually being integrated into risk prediction and management systems. In this context, AI-based techniques have the potential to drive risk-guided management strategies for both primary and secondary prevention in the near future.
